# Complications and Revision Patterns After 3D-Printed Vertebral Body Replacement for Spinal Tumors: A Systematic Review and Critical Appraisal

**DOI:** 10.3390/jcm15093447

**Published:** 2026-04-30

**Authors:** Viktor Aleinikov, Talgat Kerimbayev, Daryn Borangaliyev, Galymzhan Kadirbekov, Zhandos Tuigynov, Nurzhan Abishev, Daniyar K. Zhamoldin, Meirzhan Oshayev, Yergen Kenzhegulov, Yermek Urunbayev, Zhanibek Baiturlin, Makar Solodovnikov, Serik Akshulakov

**Affiliations:** 1 Department of Spinal Neurosurgery and Peripheral Nervous System Pathology, National Center for Neurosurgery, Astana 010000, Kazakhstan; doctor.aleynikov@gmail.com (V.A.); prof.kerimbayev.neurosurg@gmail.com (T.K.); kadirbekovgalymzan@gmail.com (G.K.); dr.tuigynov@gmail.com (Z.T.); dr.nb.abishev@gmail.com (N.A.); dockfreud@gmail.com (D.K.Z.); dr.neurosurgeon@mail.ru (M.O.); dr.kenzhegulov@gmail.com (Y.K.); doctor.urunbayev@gmail.com (Y.U.); solodovnikov.makar84@gmail.com (M.S.); 2 Department of Radiology and Radiosurgery, National Center for Neurosurgery, Astana 010000, Kazakhstan; oil.zhan64@gmail.com; 3 National Center for Neurosurgery, Astana 010000, Kazakhstan; s.akshulakov@gmail.com

**Keywords:** 3D printing, vertebral body replacement, spinal tumors, complications, revision surgery, systematic review

## Abstract

**Background**: Three-dimensional (3D)-printed vertebral body replacement (VBR) and artificial vertebral body (AVB) implants are increasingly used for anterior column reconstruction after spinal tumor resection. However, the available evidence on complications remains limited, heterogeneous, and methodologically inconsistent. This systematic review aimed to synthesize reported complications, revision patterns, and mechanical outcomes of 3D-printed VBR/AVB implants in spinal oncology and to critically appraise the quality of the available clinical literature. **Methods**: This systematic review was conducted in accordance with PRISMA 2020. PubMed/MEDLINE, Embase, and the Cochrane Library were searched from 1 January 1980 to 26 February 2026. Eligible studies included clinical series and cohort studies reporting extractable complication and/or revision data in patients who underwent spinal tumor resection followed by reconstruction with a 3D-printed VBR/AVB implant. Methodological quality was assessed using the Joanna Briggs Institute Critical Appraisal Checklist for Case Series. Due to substantial clinical and methodological heterogeneity, a structured narrative synthesis was performed. **Results**: Eleven studies comprising 217 analyzable 3D-printed reconstructions were included. Most were retrospective single-center series and showed marked heterogeneity in tumor histology, spinal level, implant strategy, follow-up duration, and complication definitions. Because adverse-event reporting was inconsistent across studies, no pooled overall complication rate was calculated. Reported perioperative non-mechanical complications included neurological deterioration, cerebrospinal fluid- or dural-related events, wound infection, pleural effusion, pneumonia, and vascular injury. Mechanical implant failure appeared relatively uncommon, although radiographic subsidence was variably defined and inconsistently reported. Implant mismatch and hardware-related problems were infrequent but clinically relevant, particularly with prefabricated or off-the-shelf devices. Revision procedures were most commonly associated with wound complications, clinically significant subsidence, hardware failure, or tumor recurrence. Overall study quality was limited by retrospective designs, small sample sizes, and non-standardized outcome reporting. **Conclusions**: Current evidence suggests that 3D-printed VBR/AVB implants are a feasible option with encouraging mechanical performance for spinal reconstruction after tumor resection. Most reported adverse events appear to reflect the complexity of oncologic spine surgery rather than device-specific failure alone. However, the available evidence remains low level and heterogeneous. Larger multicenter comparative studies with standardized outcome definitions and longer follow-up are needed to better define the clinical value and durability of 3D-printed vertebral reconstruction in spinal oncology.

## 1. Introduction

Spinal tumors, including primary malignant neoplasms and metastatic lesions, frequently cause vertebral body destruction, mechanical instability, severe pain, and neurological compromise. In carefully selected patients, surgical management may involve radical en bloc resection or total en bloc spondylectomy (TES), followed by reconstruction of the anterior spinal column to restore structural integrity and permit mobilization [1,2]. Despite advances in spinal oncology and refinements in resection techniques, restoration of spinal stability after extensive tumor removal remains technically demanding and is associated with a substantial risk of both early and late complications [2,3,4].

Traditionally, anterior column reconstruction after vertebral resection has relied on titanium mesh cages (TMCs) and expandable implants. However, these devices have been associated with clinically relevant mechanical complications, including subsidence, instrumentation failure, and loss of alignment or correction, particularly after multilevel resections and in junctional regions of the spine, where biomechanical demands are greatest [2,5]. Such failures may result in progressive deformity, compromised construct durability, reoperation, and inferior functional recovery, making the mechanical reliability of reconstruction a central issue in oncologic spine surgery [6].

Recent advances in additive manufacturing have enabled the clinical use of three-dimensional (3D)-printed vertebral body replacement (VBR) implants, including patient-specific porous titanium constructs designed for anterior column reconstruction. These implants are intended to provide improved anatomical conformity to the post-resection defect, a larger contact surface with the adjacent endplates, and potentially more favorable conditions for load transfer and osseointegration [5,7]. Early clinical series and comparative observations have reported encouraging functional, radiographic, and oncological outcomes following the use of 3D-printed implants in patients with spinal tumors [8].

Nevertheless, the evidence regarding complications after 3D-printed VBR or artificial vertebral body (AVB) reconstruction remains limited and heterogeneous. Most published studies are retrospective case series with small sample sizes, and complications are often reported as secondary outcomes without standardized definitions or uniform reporting frameworks [5,7,8]. In particular, diagnostic thresholds for subsidence, definitions of instrumentation failure, classification of mechanical events, and indications for revision surgery vary substantially across studies, limiting comparability and complicating evidence synthesis [9]. In addition, formal methodological appraisal of the available literature using validated instruments, including the Joanna Briggs Institute (JBI) tools, has been inconsistently performed, thereby reducing confidence in the robustness and clinical interpretability of the published evidence [10].

Accordingly, a structured synthesis of the frequency, spectrum, and clinical relevance of complications associated with 3D-printed vertebral reconstruction in spinal oncology is needed, with particular attention to mechanical events and revision procedures. The aim of the present systematic review was to characterize the complication profile of 3D-printed VBR/AVB implants used after spinal tumor resection and to perform a formal methodological appraisal of the included studies using the Joanna Briggs Institute (JBI) Critical Appraisal Checklist [10]. The findings were interpreted in light of the clinical complexity of oncologic spine surgery, heterogeneity in study design, and variability in follow-up duration.

## 2. Materials and Methods

### 2.1. Reporting Guidance

This systematic review was conducted and reported in accordance with PRISMA 2020. The completed PRISMA 2020 checklist and PRISMA flow diagram are provided in the Supplementary Materials [11]. The review was not prospectively registered, and no publicly accessible review protocol was prepared.

### 2.2. Data Sources and Search Strategy

A comprehensive literature search was performed in PubMed/MEDLINE, Embase, and the Cochrane Library from 1 January 1980 to 26 February 2026, with the final search conducted on 26 February 2026. The search strategy combined controlled vocabulary terms, including MeSH and Emtree where applicable, with free-text keywords related to three core domains: spinal tumors, three-dimensional (3D) printing and 3D-printed vertebral body replacement, and en bloc resection or total en bloc spondylectomy. The core concept structure included combinations of terms such as: (spine OR spinal) AND (tumor OR neoplasm OR spinal tumor) AND (3D printing OR three-dimensional printing OR 3D-printed OR artificial vertebral body OR AVB OR vertebral body replacement OR VBR) AND (total en bloc spondylectomy OR TES OR spondylectomy OR en bloc). The strategy was adapted to the syntax and indexing system of each database. The full electronic search strategies for all databases are presented in Supplementary Table S1.

To reduce the risk of missing eligible studies, we additionally screened the reference lists of all included articles and performed forward citation searching of key index studies. No study design filters were applied during the search stage; eligibility was determined during screening according to prespecified criteria. All retrieved records were exported to Zotero (Corporation for Digital Scholarship, Vienna, VA, USA) for reference management. Duplicate records were identified and removed using Zotero’s “Duplicate Items” function before title and abstract screening.

### 2.3. Eligibility Criteria

We included clinical studies enrolling patients with primary malignant and/or metastatic spinal tumors who underwent anterior column reconstruction using a 3D-printed vertebral body replacement (VBR) or artificial vertebral body (AVB) implant after tumor resection, and that reported extractable data on complications and/or revision surgery. Eligible study designs included retrospective or prospective cohort studies, comparative studies in which outcome data for the 3D-printed VBR/AVB subgroup could be extracted separately, and case series including at least four patients. This threshold was selected pragmatically to exclude isolated case reports and very small technical notes while retaining limited but clinically informative evidence in this emerging field, where the available literature remains sparse and heterogeneous.

We excluded: (1) case reports or case series including fewer than four patients and/or not providing extractable complication data; (2) studies in which 3D printing was used only for anatomical models, surgical guides, or preoperative planning without implantation of a 3D-printed VBR/AVB; (3) preclinical, biomechanical, finite-element, or other non-clinical studies without patient outcomes; (4) studies without extractable data on complications or revision procedures; and (5) duplicate or overlapping reports, in which case the most complete and informative publication was retained. Non-English publications were excluded when full-text assessment and reliable data extraction were not feasible.

### 2.4. Selection Process

**Figure 1:**
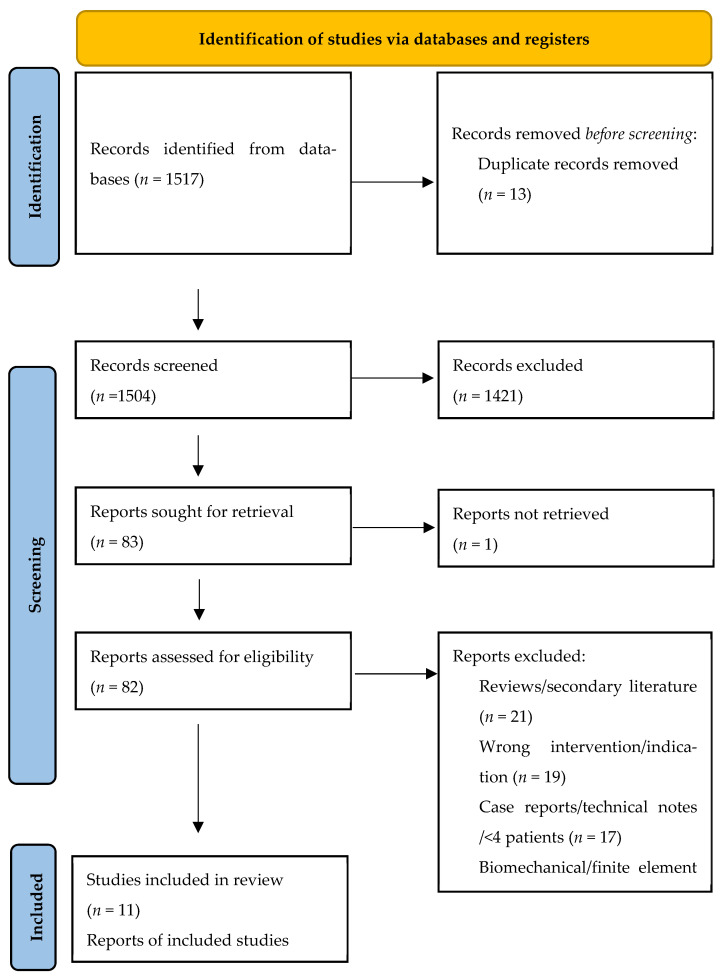
PRISMA flowchart.

Two reviewers independently screened titles and abstracts to identify potentially eligible studies. Full texts of all records considered relevant after initial screening were then assessed independently and in duplicate against the predefined eligibility criteria. Disagreements were resolved by discussion and consensus; when necessary, a third reviewer acted as arbiter. The study selection process is summarized in a PRISMA flow diagram (Figure 1).

### 2.5. Data Extraction and Data Items

Data were extracted independently by two reviewers using a standardized, pilot-tested extraction form. The following variables were collected where available: study characteristics (study design, center, study period, sample size, and follow-up duration); tumor-related characteristics (primary versus metastatic disease and spinal level); surgical details (extent of resection, posterior fixation strategy, and involvement of junctional regions); implant-related characteristics (patient-specific versus modular or standardized additively manufactured VBR, implant material, and porous architecture when reported); and outcome data relevant to postoperative complications and revision surgery.

### 2.6. Outcomes and Definitions

The primary outcome of interest was the pattern of reported postoperative complications associated with spinal tumor resection followed by reconstruction using a 3D-printed VBR/AVB. Secondary outcomes included revision surgery and mechanical reconstruction-related events. Because outcome definitions were not fully standardized across the included studies, complications were extracted and classified according to the terminology used in the original reports and then grouped into clinically relevant domains for synthesis. These domains included neurological complications, cerebrospinal fluid (CSF)-related or dural events, wound-related and infectious complications, cardiopulmonary complications, mechanical or implant-related complications, and revision procedures. When reported, the timing, clinical context, and management of complications were also recorded.

### 2.7. Data Synthesis

Given the substantial clinical and methodological heterogeneity across studies, including differences in study design, patient populations, tumor histology, spinal level, surgical technique, fixation strategy, follow-up duration, and definitions of complications, formal quantitative pooling was not considered appropriate. Quantitative synthesis was considered only when outcome definitions, clinical context, ascertainment, and follow-up structure were judged sufficiently comparable across studies. Because these conditions were not met, a structured descriptive narrative synthesis of complication patterns was performed.

Reported adverse events were summarized at the study level and organized into predefined clinical domains: neurological complications, CSF-related or dural events, wound-related and infectious complications, cardiopulmonary complications, mechanical or implant-related complications, and revision surgery. Particular attention was given to distinguishing complications likely related to the complexity of tumor resection and oncologic spinal reconstruction from those directly attributable to the implanted 3D-printed vertebral body replacement when such differentiation was possible from the source reports.

### 2.8. Subgroup Framework and Handling of Heterogeneous Definitions

To support structured interpretation of heterogeneity, findings were explored descriptively according to tumor type, spinal region, implant strategy, and the apparent attribution of adverse events as predominantly procedure-related, predominantly implant-related, or mixed/unclear. These subgroup explorations were descriptive only and were not intended to support formal pooled between-group comparisons.

Complications were extracted as originally reported and then mapped to broader clinical categories for synthesis. When attribution, timing, or severity thresholds were unclear or mixed across studies, events were retained without reviewer-driven reclassification and interpreted conservatively in the narrative synthesis.

### 2.9. Quality Assessment

Methodological quality and the completeness of complication reporting were appraised using the Joanna Briggs Institute (JBI) Critical Appraisal Checklist for Case Series [10]. This tool was selected because the evidence base consisted predominantly of uncontrolled surgical series, and the aim of the review was to evaluate the reporting quality of extracted 3D-printed VBR/AVB clinical datasets rather than to estimate comparative treatment effects. Two reviewers independently assessed each included study. Disagreements were resolved by consensus, and a third reviewer adjudicated when required. Item-level judgments (Yes/No/Unclear/Not applicable) are presented in Table 1 and summarized visually in a traffic-light plot provided in the Supplementary Materials (Figure S1).

## 3. Results

### 3.1. Study Selection

The database search identified 1517 records across PubMed/MEDLINE, Embase, and the Cochrane Library. After removal of 13 duplicates, 1504 unique records underwent title and abstract screening, of which 1421 were excluded. Eighty-three full-text reports were sought for retrieval; one report could not be obtained. Thus, 82 full-text articles were assessed for eligibility, and 71 were excluded for the following reasons: reviews or secondary literature (*n* = 21), wrong intervention or non-eligible indication (*n* = 19), case reports or technical notes with fewer than four patients (*n* = 17), biomechanical or finite-element studies without clinical outcomes (*n* = 11), engineering- or materials-focused studies without clinical data (*n* = 2), and studies of 3D-printed anatomical models without implantation of a vertebral body replacement device (*n* = 1). Eleven studies met the inclusion criteria and were included in the review (Figure 1).

### 3.2. Study Characteristics

The 11 included studies provided data on 217 analyzable 3D-printed vertebral body replacement/artificial vertebral body (3D-VBR/AVB) reconstructions after spinal tumor resection. All studies were single-center clinical series. Most were retrospective in design, one was a prospective observational case series, and two were comparative cohort studies from which only the 3D-printed reconstruction arm was extracted for synthesis. The included cohorts were heterogeneous with respect to tumor histology, spinal level, implant strategy, fixation constructs, and follow-up framework. Thoracic and thoracolumbar reconstructions predominated, whereas one study focused specifically on upper cervical (C2) tumors. Implant strategies included patient-specific/custom devices, modular systems, and prefabricated or off-the-shelf implants, typically used in conjunction with long-segment posterior fixation. Study-level characteristics are summarized in Table 2.

### 3.3. Complication Taxonomy and Interpretation of Reported Outcomes

Because the included studies differed substantially in the definitions, timing, and reporting structure of adverse events, no pooled “overall complication rate” was calculated. Some studies reported the number of patients with at least one complication, whereas others reported only event counts, selected complication categories, radiographic findings, revision procedures, or mortality during follow-up. Accordingly, reported proportions were interpreted descriptively and are not directly comparable across cohorts.

For structured synthesis, reported outcomes were classified into four prespecified domains. First, perioperative non-mechanical complications included neurological events, cerebrospinal fluid (CSF)-related or dural events, wound-related or infectious complications, and cardiopulmonary, vascular, or medical adverse events reported intraoperatively, postoperatively, or during the index hospitalization, as described by the original study authors. Second, mechanical or reconstruction-related outcomes included implant subsidence, implant mismatch, instrumentation failure, prosthesis migration or dislodgement, prosthesis fracture, and screw loosening or breakage. Because definitions of subsidence were not uniform across studies, no harmonized threshold was imposed retrospectively; instead, subsidence was recorded according to the original study definition, including explicit radiographic cutoffs when provided. Third, revision procedures were recorded separately and classified according to the reported indication, including wound complications, mechanical failure, clinically relevant subsidence, junctional complications, hardware-related problems, or tumor recurrence/progression requiring implant removal or reoperation. Fourth, mortality was documented separately as perioperative mortality or disease-related mortality during follow-up whenever this distinction was possible.

Intraoperative events were included only when the original authors explicitly reported them as complications or clinically relevant adverse events. Accordingly, events such as pleural rupture were retained in the descriptive synthesis but interpreted as procedure-related intraoperative adverse events rather than postoperative implant-specific complications. Likewise, deaths attributable to systemic oncologic progression during follow-up were recorded as study outcomes when reported but were not classified as implant-related complications. Because follow-up was reported inconsistently across studies, including both mean and median values and, in some comparative cohorts, not always extractable for the 3D arm alone, no pooled summary follow-up estimate was calculated.

**Table 2:** Study characteristics of included studies.

Study	Design/Period	3D Cohort Analyzed, *n*	Tumor Type	Spinal Region/Level	3D Implant Strategy	Material/Porous Features	Posterior Fixation Strategy	Age, Years	Follow-Up, Months
Hu X 2022 [12]	Retrospective single-center case series (2018–2021)	8 enrolled (6 implanted)	Primary 6/8 (75.0%); metastatic 1/8 (12.5%); secondary kyphosis/revision 1/8 (12.5%)	Thoracic 4; cervicothoracic 1; upper thoracic 1	Patient-specific custom 3D-printed AVB/VBR	Ti6Al4V, SLM; porous endplates 70 ± 10%; pores 600–800 µm	Long-segment posterior fixation ≥2 levels above/below	Median 34 (22–53)	Median 11.5
Zhou H 2022 [14]	Retrospective observational single-center study (2016–2019)	23	Primary 18/23 (78.3%); metastatic 5/23 (21.7%)	Thoracolumbar; TES 20, sagittal resection 3	Custom 10; off-the-shelf 13	Ti6Al4V, EBM; pores 600 ± 200 µm; porosity 50–80%	Pedicle screws + CoCr rods + transverse connectors	41 (17–71)	Median 37 (24–58)
Ji J 2025 [15]	Retrospective single-center case series (2016–2023)	43	Primary malignant 12/43 (27.9%); metastatic 31/43 (72.1%)	Cervical 3; thoracic 34; lumbar 6; mostly single-level (36)	3D-printed artificial vertebra	Ti6Al4V, EBM; porosity 80%; pore 800 ± 200 µm	Posterior pedicle screw–rod fixation in all; two-stage surgery	58 (15–76)	Mean 10.9 (3–31)
Aleinikov VG 2025 [16]	Retrospective single-center case series (2019–2023)	4	Primary 3/4 (75.0%); metastatic 1/4 (25.0%)	Thoracic (Th7, Th9, Th10 ×2)	Patient-specific fully personalized modular implant	Ti6Al4V powder; trabecular-like lattice architecture	Transpedicular fixation 2 levels above/2 below	49.8 ± 15.7	24
Girolami 2018 [17]	Prospective observational case series, single center (2015–2017)	13	Primary 8/13 (61.5%); metastatic 5/13 (38.5%)	Thoracic 6; lumbar 7; T10–L2 junction involved in 11	Patient-specific BiomimeTiC titanium prosthesis	Ti6Al4V, EBM; lattice + cortical-like shell	Single-level: 2–3 above + 1–2 below; double-level: 2–3 above + 2–3 below	Mean 47 (18–73)	Mean 10 (2–16)
Sun Z 2022 [13]	Retrospective single-center case series (2017–2018)	8	Primary 6/8 (75.0%); metastatic 2/8 (25.0%)	Thoracic 6; cervicothoracic 2; multilevel TES	Patient-specific custom AVB	Ti6Al4V, EOS M280; porous trabecular-like structure; pores 600–800 µm; porosity 70–80%	NR	34.5 (22–51)	24 (18–40)
Wang X 2024 [8]	Retrospective single-center case series (2017–2020)	14	Primary 4/14 (28.6%); metastatic 10/14 (71.4%)	Thoracolumbar; TES single-segment 10, two-segment 2, three-segment 2	Prefabricated 9; custom 5	Titanium alloy, EBM; porosity 80%; pore 800 ± 200 µm	Posterior pedicle screws in all; usually 2 pairs above/below; extended when needed	54.1 ± 17.1 (15–73)	Mean 19.9 ± 9.5 (7–43)
Tang X 2021 [18]	Retrospective single-center case series (2016–2019)	27	Primary 23/27 (85.2%); metastatic 4/27 (14.8%)	Thoracolumbar; multilevel TES (2–6 levels)	3D-printed modular vertebral prosthesis	Ti6Al4V; modular; porous endplate surface + conical projections	Posterior instrumentation 2–3 above/below; posterior-only 11, combined 16	42 (15–72)	Mean 22 (12–41)
Hu P 2022 [20]	Retrospective comparative single-center cohort (2009–2020); extracted 3D arm	18	Primary only: chordoma 8/18 (44.4%), giant cell tumor 8/18 (44.4%), paraganglioma 1/18 (5.6%), Ewing sarcoma 1/18 (5.6%)	C2 (upper cervical)	Patient-specific custom 3D-printed AVB	Ti6Al4V, EBM; porosity 50–80%; pore 600 ± 200 µm; wire 550 ± 200 µm	Staged posterior + anterior; occipital or C1–C4/C5 screw–rod fixation	38.2 ± 3.8	NE for AVB arm
Hu J 2023 [19]	Retrospective single-center cohort (2017–2022)	51	Primary 33/51 (64.7%); metastatic 18/51 (35.3%)	Thoracic 35; lumbar 16	Custom 10; off-the-shelf 41	Ti6Al4V; EBM (Arcam Q10plus) for customized implants	NR	41.9 ± 16.0	Median 21 (7–57)
Cao Y 2023 [21]	Retrospective comparative single-center cohort (2019–2021); extracted 3D arm	10	Metastatic only: breast 3/10 (30.0%), lung 2/10 (20.0%), prostate 2/10 (20.0%), stomach 2/10 (20.0%), colorectal 1/10 (10.0%)	Thoracolumbar	Patient-specific 3D-printed auto-stable AVB	Porous structure: pore 700 ± 80 µm; wire 300 ± 100 µm; porosity 73%	Posterior approach; pedicle screws with transverse connectors; compression seating	55.4 ± 14.3	Median 21.8 (12–38)

Note. AVB, artificial vertebral body; VBR, vertebral body replacement; TES, total en bloc spondylectomy; NR, not reported; NE, not extractable. For studies with mixed enrollment and implantation denominators, both are shown explicitly. In Hu X 2022, 8 patients were enrolled, 6 underwent successful implantation of the patient-specific 3D-printed AVB/VBR, and 2 required intraoperative conversion because of implant mismatch. For comparative cohorts, arm-specific follow-up was reported only when clearly extractable from the original article.

### 3.4. Perioperative Non-Mechanical Complications

Perioperative non-mechanical complications were reported in most included studies, although the depth and granularity of reporting varied considerably. At the individual-study level, reported complication burden ranged from no reported complications in a small personalized-implant series to high perioperative morbidity in larger thoracolumbar TES cohorts. However, these differences should be interpreted cautiously, as they likely reflect variation in cohort size, case complexity, surgical extent, and reporting methodology rather than directly comparable complication risks. Reported perioperative complications are summarized in Table 3.

### 3.5. Neurological Complications

Neurological complications were variably defined and inconsistently reported. Hu J et al. [19] documented postoperative neurological deterioration in 5/51 patients, whereas Zhou et al. [14] reported five sensorimotor disorder events. Tang et al. described one case of monoplegia in the setting of planned root sacrifice and two cases of postoperative neurological deterioration, both of which reportedly improved during follow-up [18]. In the metastatic thoracolumbar cohort reported by Cao et al., hypaesthesia was described in 1/10 patients [21]. Sun et al. additionally reported intercostal neuralgia in 2/8 patients [13]. Overall, neurological morbidity was observed across several studies, but inconsistent definitions and mixed attribution limited direct cross-study comparison.

### 3.6. CSF-Related and Dural Events

CSF-related or dural complications were among the most consistently reported perioperative adverse events. In the Hu X series, one complication was reported in the successfully implanted subgroup (1/6), although the original cohort comprised eight candidates, two of whom required intraoperative conversion because of implant mismatch [12]. Sun et al. reported CSF leakage in 2/8 patients [13], Cao et al. reported CSF leakage in 4/10 [21], Zhou et al. [14] reported four CSF leak events, and Wang et al. reported dural injury/CSF leak in 3/14 patients [8]. Taken together, these findings suggest that CSF-related morbidity is a recurrent perioperative issue in complex oncologic spinal reconstruction, particularly in thoracic and thoracolumbar procedures involving extensive exposure and resection.

### 3.7. Wound-Related and Infectious Complications

Wound-related morbidity was particularly prominent in the larger thoracolumbar series. Hu J et al. [19] reported deep wound infection in 9/51 patients and identified wound-related complications as a major indication for reoperation. Zhou et al. [14] reported three wound infection events, Tang et al. [18] reported one wound infection requiring debridement, and Wang et al. [8] described delayed wound healing in 1/14 patients. By contrast, no wound complications were reported in the small personalized-implant series by Aleinikov et al. [16], although that cohort included only four patients and should therefore be interpreted cautiously.

### 3.8. Cardiopulmonary, Vascular, and Medical Complications

Cardiopulmonary and pleural complications were recurrent, particularly in thoracic resections. Hu J et al. [19] reported pleural effusion in 10/51 patients, while Zhou et al. [14] documented six pleural effusion events together with isolated systemic complications such as deep venous thrombosis, heart failure, and delirium. Wang et al. [8] reported intraoperative pleural rupture in 6/14 patients and postoperative pleural effusion requiring drainage in 2/14. Tang et al. [18] described pneumonia or respiratory failure requiring reintubation in five patients, including one perioperative death, and additionally reported two major vascular injuries. Overall, these events appear to reflect the complexity of thoracic and multilevel oncologic resections rather than device-specific failure alone.

### 3.9. Mechanical/Reconstruction-Related Outcomes

Mechanical outcomes were defined inconsistently across studies and should be interpreted separately from perioperative non-mechanical complications. Some cohorts used explicit radiographic thresholds for subsidence, whereas others reported the absence of “subsidence” or “failure” without a detailed operational definition. Girolami et al. [17] reported radiographic subsidence in 11/12 evaluable patients, but most cases were described as clinically minor; only one patient underwent revision for subsidence, and the authors suggested that distal junctional mechanics rather than true prosthesis failure may have contributed. Wang et al. [8] reported prosthetic subsidence ≥2 mm in 3/14 patients, all in the prefabricated implant subgroup, with one case of secondary screw loosening and no rod failure, prosthesis fracture, dislodgement, or screw breakage/pullout. Tang et al. [18] reported asymptomatic subsidence in 10 patients, without evidence of internal fixation failure or prosthesis dislocation among patients with at least one year of follow-up. Hu J et al. reported no prosthesis subsidence but did document instrumentation failure in 1/51 patients [19]. In Ji et al., no prosthesis subsidence or screw loosening was reported, although mortality during follow-up was driven by systemic metastatic disease rather than construct failure [15]. Mechanical/reconstruction-related outcomes are summarized in Table 4.

### 3.10. Implant Mismatch and Device-Related Fit Issues

Implant mismatch was reported primarily in association with non-custom or prefabricated devices. In the Hu X study, two of eight candidates required intraoperative conversion because the patient-specific implant could not be seated as planned [12]. In the Hu J cohort, mismatch events were confined to off-the-shelf implants [19]. These observations suggest that implant fit remains a clinically relevant issue in complex vertebral reconstruction, although the underlying mechanism and clinical consequences were not reported uniformly across studies.

### 3.11. Revision Procedures

Revision procedures were reported inconsistently and arose from multiple causes rather than a single dominant failure pattern. Reoperations were performed for wound complications, clinically significant subsidence, hardware-related problems, junctional complications, and, in some cases, tumor recurrence necessitating implant removal. Hu J et al. reported at least one reoperation in 13/51 patients [19]. Girolami et al. reported one revision for subsidence, one revision for distal junctional kyphosis, and one implant removal due to local recurrence [17]. In the C2 cohort reported by Hu P et al., a hardware-related problem at follow-up was attributed to C1 screw malposition, while the AVB itself remained stable [20]. These findings support separating revision procedures by indication rather than treating all revisions as equivalent markers of implant failure.

### 3.12. Mortality

Mortality should be interpreted separately from implant-related outcomes. Perioperative mortality was uncommon but was reported in one multilevel thoracolumbar series in the setting of severe postoperative respiratory complications. By contrast, deaths during follow-up in metastatic cohorts reflected underlying oncologic disease progression rather than failure of the 3D-printed vertebral reconstruction itself. Where possible, perioperative mortality and disease-related mortality during follow-up were recorded separately in Table 4.

**Table 4:** Mechanical outcomes, revision procedures, and mortality.

Study	Subsidence	Implant Mismatch	Instrumentation Failure	Implant Migration/Fracture/Dislodgement	Revision Procedures	Mortality (Perioperative/Follow-Up)	Notes
Hu X 2022 [12]	0/6 implanted (0%) reported	2/8 intraoperative mismatch → conversion	0/6 implanted (0%)	0/6 implanted (0%)	2/8 candidates (25.0%) intraoperative conversion	NR	Conversions reflect implantation failure rather than late mechanical failure
Zhou H 2022 [14]	NR	NR	Rod fracture/failure 2/23 (8.7%)	0/23 (0%) prosthesis migration reported	2/23 (8.7%) revision for repeated rod breakage	0/2 tumor-related deaths during follow-up	Instrumentation failure occurred in patients with marked subsidence (8.47 and 3.69 mm)
Ji J 2025 [15]	0/43 reported	NR	0/43 (0%) screw loosening reported	0/43 (0%) prosthesis migration/fracture reported	NR	0/43 (0%) perioperative; 8/43 (18.6%) follow-up deaths	Mortality reflects oncologic progression rather than device-related failure
Aleinikov VG 2025 [16]	0/4 (0%)	0/4	0/4 (0%) fixation failure	0/4 (0%) prosthesis migration/fracture	NR	NR	CT showed no implant-related complications
Girolami 2018 [17]	11/12 (91.7%) minor radiographic; 1/12 (8.3%) clinically relevant	NR	0/12 (0%) breakage reported	0/12 (0%) migration reported	1/12 (8.3%) revision for subsidence; 1/13 (7.7%) implant removal for local recurrence; 1/13 (7.7%) DJK revision	NR	Subsidence denominator reflects available radiographic follow-up
Sun Z 2022 [13]	0/8 (0%) sinking/subsidence reported	NR	NR	NR	NR	NR	The prosthesis matched well and fixation remained reliable during follow-up
Wang X 2024 [8]	≥2 mm in 3/14 (21.4%), all prefabricated	0/14 (0%) reported	Screw loosening secondary to subsidence 1/14 (7.1%)	0/14 (0%) rod failure, prosthesis fracture/dislodgement, or screw breakage/pullout reported	NR	NR	Mechanical events concentrated in prefabricated group
Tang X 2021 [18]	10/26 (38.5%) asymptomatic radiographic; mean 1.8 ± 1.0 mm	NR	0/26 (0%) major instrumentation failure reported	0/26 (0%) migration/breakage reported	1/27 (3.7%) debridement for wound infection	1/27 (3.7%) perioperative death	Radiographic subsidence was asymptomatic in available follow-up
Hu P 2022 [20]	0/18 (0%) AVB stable; no subsidence reported	0 reported	1/18 (5.6%) hardware problem at follow-up due to C1 screw malposition	0/18 (0%) AVB instability reported	NR	0/18 (0%) perioperative; 0/18 (0%) follow-up reported	Hardware issue reflected screw malposition rather than AVB failure
Hu J 2023 [19]	0/51 (0%) prosthesis subsidence reported	2/51 (3.9%), both off-the-shelf lumbar cases	1/51 (2.0%)	Displacement of prosthesis 1/51 (2.0%); fracture 0/51 (0%) reported	13/51 (25.5%) at least one reoperation; wound-related complications were the most common indication	0/51 (0%) perioperative; 5/51 (9.8%) follow-up deaths	Three local recurrences were reported; five patients died by final follow-up
Cao Y 2023 [21]	NR	NR	NR	NR	NR	NR	Mechanical outcomes were not clearly described in the extracted 3D arm

Note. Mechanical outcomes were recorded separately from perioperative non-mechanical complications. Radiographic subsidence was not considered equivalent to clinically significant implant failure unless associated with symptoms, progression, screw loosening, loss of correction, or revision. Revision procedures are reported according to the indication described in the source study. Where clearly extractable, categorical data are presented as n/N (%). NR = not reported or not reliably extractable from the study report. Intraoperative conversion due to implant mismatch was retained as a mechanical implementation outcome.

## 4. Discussion

The present systematic review synthesizes the currently available clinical evidence regarding complications associated with 3D-printed vertebral body replacement (VBR/AVB) following spinal tumor resection. Across 11 clinical studies including 217 patients, the literature demonstrates that reconstruction with additively manufactured implants is technically feasible and generally associated with acceptable complication profiles in the setting of complex oncologic spine surgery. However, complication reporting across studies remains heterogeneous, with substantial variation in definitions, follow-up duration, and outcome reporting. The results of this review suggest that while mechanical implant failure appears relatively uncommon, a broad spectrum of perioperative complications—ranging from neurological and dural events to systemic cardiopulmonary complications—remains inherent to radical spinal tumor surgery, particularly in procedures involving TES. These findings highlight the importance of interpreting implant-related outcomes within the broader context of high-complexity oncologic spine procedures, rather than evaluating reconstruction devices in isolation.

An important question is whether 3D-printed vertebral implants provide measurable advantages over conventional reconstruction techniques, particularly titanium mesh cages (TMC), which have historically been the standard method for anterior column reconstruction [22]. Conventional cages have been associated with relatively high rates of implant subsidence and loss of correction, especially in multilevel resections or in biomechanically demanding spinal regions [21,23]. In contrast, additively manufactured implants offer several theoretical advantages, including patient-specific anatomical matching, increased endplate contact area, and porous structures designed to promote osseointegration [24]. These features may improve load distribution and potentially reduce subsidence risk compared with standard cages [19,23]. However, direct comparative clinical evidence remains limited. Most available studies are small retrospective series, and only a few cohorts directly compare 3D-printed implants with conventional cages [16,21,25]. Consequently, although early clinical results are encouraging, further comparative and multicenter studies with standardized outcome reporting are required to determine whether 3D-printed vertebral implants provide a clear biomechanical or clinical advantage over traditional reconstruction methods.

### 4.1. Mechanical Complications

Mechanical stability is a critical determinant of successful reconstruction after spinal tumor resection, particularly following total en bloc spondylectomy (TES), where extensive vertebral removal and long posterior fixation constructs significantly alter spinal biomechanics. In the present review, mechanical outcomes were reported heterogeneously, but several consistent trends can be identified.

Subsidence reporting varied widely across studies, reflecting the absence of standardized definitions. Some authors applied explicit radiographic thresholds, whereas others simply reported the absence of subsidence without defining measurement criteria. Excluding the outlier series of Girolami et al., where minor radiographic subsidence occurred in 11/12 patients (92%) but required revision in only 1/12 (8.3%), the remaining studies reported relatively low subsidence rates ranging from 0% to 21.4%. Wang et al. observed subsidence ≥2 mm in 3/14 patients (21.4%), notably all in the prefabricated implant subgroup, whereas no subsidence occurred among customized implants. Similarly, Ji et al. and Hu J et al. reported no prosthesis subsidence, and Aleinikov et al. identified no radiographic evidence of implant-related mechanical complications during follow-up.

Instrumentation failure appeared uncommon throughout the available literature. Only one larger cohort reported this complication, with Hu J et al. documenting instrumentation failure in 1/51 patients (2.0%), while other studies reported no rod fracture, screw loosening, or implant breakage. In addition, implant mismatch emerged as a specific mechanical concern. Hu X et al. reported 2/8 intraoperative mismatches requiring conversion, and Hu J et al. observed implant mismatch in 2/51 patients (3.9%), predominantly associated with off-the-shelf implants.

Several biomechanical mechanisms may explain the favorable mechanical performance reported in many series. Patient-specific implants allow improved endplate conformity, increasing the contact surface between the implant and adjacent vertebrae and potentially reducing localized stress concentrations. In addition, porous titanium architectures facilitate osteointegration, enabling progressive biological fixation and improved load transfer. However, interpretation of these results remains limited by heterogeneous subsidence definitions, inconsistent reporting of mechanical endpoints, and relatively short follow-up periods, which restrict evaluation of late complications such as junctional failure or delayed implant collapse.

### 4.2. Neurological and Dural Complications

Neurological and dural complications represented another major category of adverse events across the included studies. The most frequently reported complications were cerebrospinal fluid (CSF) leakage, dural injury, and postoperative neurological deterioration.

CSF leakage was reported across multiple cohorts: 1/6 patients (16.7%) in Hu X et al., 2/8 (25%) in Sun et al., 4/10 (40%) in Cao et al., and 3/14 (21.4%) in Wang et al., while Zhou et al. reported four CSF leak events. Neurological deterioration was reported in 5/51 patients (9.8%) by Hu J et al., and Zhou et al. described five sensorimotor deficit events. Tang et al. reported one case of monoplegia related to planned root sacrifice and two cases of postoperative neurological deterioration, both of which recovered.

Importantly, these complications are more plausibly associated with the tumor resection procedure itself rather than the reconstruction implant. TES and similar oncologic resections require extensive dissection around the spinal cord, dura mater, and nerve roots, frequently in the presence of epidural tumor extension or distorted anatomy. Consequently, the observed neurological and dural events likely reflect the intrinsic complexity of radical spinal tumor surgery, rather than device-specific complications related to 3D-printed vertebral implants.

### 4.3. Systemic and Thoracic Complications

Beyond local surgical complications, several studies reported systemic and thoracic postoperative events, further illustrating the multifactorial nature of morbidity following spinal tumor resection.

Thoracic and pleural complications were particularly common in cohorts dominated by thoracic or thoracolumbar resections. Hu J et al. reported pleural effusion in 10/51 patients (19.6%), while Zhou et al. documented six pleural effusion events together with isolated medical complications including deep vein thrombosis, heart failure, and delirium. Wang et al. reported intraoperative pleural rupture in 6/14 patients and pleural effusion requiring drainage in 2/14 cases. Tang et al. described pneumonia or respiratory failure requiring reintubation in five patients, including one perioperative death, and additionally reported two cases of intraoperative aortic injury.

These complications are likely related to the extensive surgical exposure and physiological stress associated with TES, including prolonged operative time, substantial blood loss, combined surgical approaches, and the anatomical proximity of thoracic resections to the pleural cavity. Accordingly, the systemic complication profile observed in the included studies should be interpreted within the broader context of high-complexity oncologic spine surgery rather than as implant-specific morbidity.

### 4.4. Methodological Limitations

The present synthesis highlights several important limitations in the existing literature on 3D-printed vertebral body replacement in spinal oncology. Most studies were retrospective single-center case series or retrospective cohorts, with only one small prospective observational series. Consequently, the available evidence remains vulnerable to selection bias and reporting bias.

In addition, the overall dataset remains limited, comprising 11 studies and 217 patients, and substantial clinical heterogeneity exists in terms of tumor histology, spinal level, surgical technique, fixation strategy, and implant design. Complication reporting was also inconsistent: some studies reported patients with ≥1 complication, whereas others reported total event counts, preventing formal pooled analysis.

Definitions of key mechanical outcomes-including subsidence, instrumentation failure, and revision indications-varied substantially across studies, and several publications did not provide operational criteria. Follow-up duration was also heterogeneous and often limited, restricting assessment of delayed mechanical complications. Finally, although some comparative cohorts exist, high-quality prospective comparative studies evaluating 3D-printed implants versus conventional reconstruction methods remain lacking.

However, the current evidence base remains limited by small retrospective cohorts, heterogeneous patient populations, inconsistent definitions of mechanical outcomes, and relatively short follow-up.

### 4.5. Clinical Implications and Future Directions

Despite these limitations, the available evidence suggests that 3D-printed vertebral body replacement represents a promising reconstructive strategy after spinal tumor resection. Potential advantages include patient-specific anatomical design, improved biomechanical load distribution, and porous implant structures that may enhance osteointegration [14,26].

However, the broader implementation of this technology beyond highly specialized centers may be constrained by several practical factors, including the need for advanced imaging, detailed preoperative planning, engineering and manufacturing support, implant production time, regulatory and quality-control requirements, and economic considerations, particularly in less-resourced settings. In addition, the currently available clinical evidence remains preliminary and is derived predominantly from experienced tertiary referral centers, which may limit the generalizability of the reported outcomes. Therefore, definitive conclusions regarding superiority over conventional reconstruction techniques cannot yet be drawn. Future research should focus on multicenter prospective cohorts, standardized definitions of mechanical complications, feasibility and cost considerations, and long-term follow-up to better characterize implant durability, revision risk, and overall clinical outcomes following 3D-printed vertebral body reconstruction in spinal oncology.

## 5. Conclusions

This systematic review indicates that 3D-printed vertebral body replacement implants represent a feasible and mechanically reliable option for anterior column reconstruction after spinal tumor resection. Across the available studies, clinically significant implant failure was uncommon, while most reported complications reflected the intrinsic complexity of radical oncologic spine surgery, including neurological, dural, wound, and cardiopulmonary events rather than device-specific problems. Although early clinical results are encouraging, the true comparative advantages of 3D-printed implants over conventional reconstruction techniques remain uncertain. Larger multicenter prospective studies with standardized outcome reporting and longer follow-up are required to define the long-term durability and clinical value of these implants in spinal oncology.

## Figures and Tables

**Table 1:** Methodological quality appraisal of included studies (3D-printed VBR/AVB arms only) using the Joanna Briggs Institute (JBI) Critical Appraisal Checklist.

Study	Design	Q1	Q2	Q3	Q4	Q5	Q6	Q7	Q8	Q9	Q10
Hu X 2022 (Int J Bioprint) [12]	Retrospective case series (3D candidates; 6/8 successful)	Y	Y	Y	N	U	Y	Y	Y	Y	Y
Sun Z 2022 (Orthop Surg) [13]	Retrospective case series (8 consecutive multilevel TES)	Y	Y	Y	Y	Y	Y	Y	Y	Y	Y
Zhou H 2022 (J Neurosurg Spine) [14]	Retrospective observational (29 enrolled; 23 analyzed)	Y	Y	Y	Y	N	Y	Y	Y	Y	Y
Ji J 2025 (Clin Spine Surg) [15]	Retrospective case series (43 malignant tumors)	Y	Y	Y	U	U	Y	Y	Y	Y	Y
Aleinikov 2025 (3D Printing in Medicine) [16]	Retrospective case series (*n* = 4; personalized implants)	Y	Y	Y	U	U	Y	Y	Y	Y	Y
Girolami 2018 (Eur Spine J) [17]	Prospective observational case series (13 non-consecutive)	Y	Y	Y	N	U	Y	Y	Y	Y	Y
Wang 2024 (BMC Musculoskelet Disord) [8]	Retrospective case series (*n* = 14 TES + 3D-AVB)	Y	Y	Y	U	U	Y	Y	Y	Y	Y
Tang X 2021 (Orthop Surg) [18]	Retrospective case series (*n* = 27 multilevel TES + modular VBR)	Y	Y	Y	U	Y	Y	Y	Y	Y	Y
Hu J 2023 (WJSO) [19]	Retrospective cohort (53 consecutive; 2 lost, *n* = 51)	Y	Y	Y	Y	N	Y	Y	Y	Y	Y
Hu P 2022 (Front Oncol)—3D arm only [20]	Comparative cohort; extracted 3D group (*n* = 18)	Y	Y	Y	U	U	Y	Y	Y	Y	Y
Cao Y 2023 (J Orthop Surg Res)—3D arm only [21]	Comparative cohort; extracted 3D group (*n* = 10)	Y	Y	Y	U	U	Y	Y	Y	Y	Y

Ratings: Y = Yes; N = No; U = Unclear/Not reported. Overall concerns reflect methodological quality of the 3D-arm reporting (not comparative effect). JBI items: Q1: Clear inclusion criteria; Q2: Condition measured in a standard, reliable way; Q3: Valid methods used to identify the condition; Q4: Consecutive inclusion; Q5: Complete inclusion; Q6: Demographics clearly reported; Q7: Clinical information clearly reported; Q8: Outcomes/follow-up results clearly reported; Q9: Presenting site(s)/clinic demographic information reported; Q10: Statistical analysis appropriate (at least descriptive where applicable).

**Table 3:** Reported perioperative non-mechanical complications.

Study	Overall Reported Complications *	Neurological	CSF/Dural	Wound/Infectious	Cardiopulmonary/Vascular/Medical	Intraoperative Events Explicitly Reported as Complications	Notes
Hu X 2022 [12]	1/8 (12.5%); implanted subgroup 1/6 (16.7%)	NR	1/8 (12.5%) postoperative CSF leak	1/8 (12.5%) associated infection	NR	NR	Single postoperative CSF leak with infection; mismatch-related conversions listed in Table 4
Zhou H 2022 [14]	18/23 patients (78.3%); 21 events	Sensorimotor disorder 5/23 (21.7%)	CSF leak 4/23 (17.4%)	Wound infection 3/23 (13.0%)	Pleural effusion 6/23 (26.1%); DVT 1/23 (4.3%); heart failure 1/23 (4.3%); delirium 1/23 (4.3%)	NR	Mixed patient- and event-level reporting
Ji J 2025 [15]	NE	NR	NR	NR	NR	NR	Study emphasized mechanical stability; perioperative complication breakdown not clearly extractable
Aleinikov VG 2025 [16]	0/4 (0%)	0/4 (0%)	0/4 (0%)	0/4 (0%)	0/4 (0%)	0/4 (0%)	No perioperative non-mechanical complications reported
Girolami 2018 [17]	NE	NR	NR	NR	NR	NR	Perioperative non-mechanical complication framework not clearly detailed
Sun Z 2022 [13]	4/8 (50.0%) perioperative complications	Intercostal neuralgia 2/8 (25.0%)	CSF leak 2/8 (25.0%)	0/8 (0%)	0/8 (0%)	0/8 (0%)	No wound or cardiopulmonary events reported
Wang X 2024 [8]	NE	NR	Dural injury/CSF leak 3/14 (21.4%)	Delayed wound healing 1/14 (7.1%)	Pleural rupture 6/14 (42.9%); pleural effusion requiring drainage 2/14 (14.3%)	Pleural rupture 6/14 (42.9%)	Overall number of patients with ≥1 complication not clearly extractable
Tang X 2021 [18]	15/27 patients (55.6%); 32 events (18 major, 14 minor)	Monoplegia 1/27 (3.7%); postoperative neurological deterioration 2/27 (7.4%)	NR	Wound infection 1/27 (3.7%)	Pneumonia/respiratory failure 5/27 (18.5%); aortic injury 2/27 (7.4%)	Aortic injury 2/27 (7.4%)	One perioperative death occurred after severe respiratory complications
Hu P 2022 [20]	5/18 (27.8%) complicated events	NE	NE	NE	NE	NE	Event categories not fully itemized for the extracted AVB arm
Hu J 2023 [19]	29/51 (56.9%); 50 perioperative events	Neurological deterioration 5/51 (9.8%)	NR	Deep wound infection 9/51 (17.6%)	Pleural effusion 10/51 (19.6%)	NR	Major/minor event counts reported separately in source study
Cao Y 2023 [21]	NE	Hypaesthesia 1/10 (10.0%)	CSF leak 4/10 (40.0%)	0/10 (0%) reported	0/10 (0%) reported	NR	Overall number of patients with ≥1 complication not clearly extractable

Note. * Reported complication proportions are descriptive and are not directly comparable across studies because source articles differed in cohort size, case mix, follow-up duration, complication definitions, and reporting structure. Values may represent either the number of patients with at least one complication or the number of complication events, depending on the original report. Where clearly extractable, categorical data are presented as *n*/N (%). NR = not reported; NE = not extractable. Intraoperative events were included only if explicitly described by the study authors as complications or clinically relevant adverse events.

## Data Availability

All data generated or analyzed during this study are included in this published article and its Supplementary Materials.

## Supplementary Materials

The following supporting information can be downloaded at https://www.mdpi.com/article/10.3390/jcm15093447/s1, File S1—Figure S1: JBI critical Appraisal Checklist (traffic-light plot); File S2—Table S1: Full Literature Search Strategy; PRISMA 2020 for Abstracts Checklist; PRISMA 2020 Checklist.

## Abbreviations

The following abbreviations are used in this manuscript:

3D	three-dimensional
AVB	artificial vertebral body
CSF	cerebrospinal fluid
DJK	distal junctional kyphosis
DVT	deep venous thrombosis
EBM	electron beam melting
HU	Hounsfield unit
SLM	selective laser melting
TES	total en bloc spondylectomy
TMC	titanium mesh cage
VBR	vertebral body replacement
