# Supplementary material for: Complications and Revision Patterns After 3D-Printed Vertebral Body Replacement for Spinal Tumors: A Systematic Review and Critical Appraisal
Source: J Clin Med. 2026 Apr 30;15(9):3447. doi: 10.3390/jcm15093447 (PMC13164235; doi:10.3390/jcm15093447)
Supplement: Supplementary file 1 [file jcm-15-03447-s001.zip › supplementary table S1.pdf]

**PubMed/MEDLINE:**	("Spinal Neoplasms"[Mesh] OR ((spine[Title/Abstract] OR spinal[Title/Abstract] OR vertebr*[Title/Abstract]) AND (tumor*[Title/Abstract] OR tumour*[Title/Abstract] OR neoplasm*[Title/Abstract] OR cancer*[Title/Abstract] OR metasta*[Title/Abstract]))) AND ("Printing, Three-Dimensional"[Mesh] OR "3D printing"[Title/Abstract] OR "3D-printed"[Title/Abstract] OR "3-dimensional printing"[Title/Abstract] OR "three-dimensional printing"[Title/Abstract] OR "three dimensional printing"[Title/Abstract] OR "additive manufacturing"[Title/Abstract] OR "patient-specific"[Title/Abstract] OR "custom-made"[Title/Abstract] OR customized[Title/Abstract]) AND ("vertebral body replacement"[Title/Abstract] OR "vertebral replacement"[Title/Abstract] OR VBR[Title/Abstract] OR "artificial vertebral body"[Title/Abstract] OR AVB[Title/Abstract] OR prosthes*[Title/Abstract] OR implant*[Title/Abstract] OR cage*[Title/Abstract]) AND ("total en bloc spondylectomy"[Title/Abstract] OR TES[Title/Abstract] OR spondylectom*[Title/Abstract] OR "en bloc"[Title/Abstract] OR vertebrectom*[Title/Abstract] OR corpectom*[Title/Abstract] OR resect*[Title/Abstract] OR reconstruct*[Title/Abstract]) AND ("1980/01/01"[Date - Publication] : "2026/02/26"[Date - Publication]).
**Cochrane Library:**	([Spinal Neoplasms] OR ((spine OR spinal OR vertebr*) NEAR/3 (tumor* OR tumour* OR neoplasm* OR cancer* OR metasta*))):ti,ab,kw AND ([Printing, Three-Dimensional] OR "3D printing" OR "3D-printed" OR "3-dimensional printing" OR "three-dimensional printing" OR "three dimensional printing" OR "additive manufacturing" OR "patient-specific" OR "custom-made" OR customized):ti,ab,kw AND ("vertebral body replacement" OR "vertebral replacement" OR VBR OR "artificial vertebral body" OR AVB OR prosthes* OR implant* OR cage*):ti,ab,kw AND ("total en bloc spondylectomy" OR TES OR spondylectom* OR "en bloc" OR vertebrectom* OR corpectom* OR resect* OR reconstruct*):ti,ab,kw.
**Embase:**	((spine OR spinal OR vertebr*):ti,ab,kw AND (tumor* OR tumour* OR neoplasm* OR cancer* OR metasta*):ti,ab,kw) AND ("3D printing":ti,ab,kw OR "3D-printed":ti,ab,kw OR "3-dimensional printing":ti,ab,kw OR "three-dimensional printing":ti,ab,kw OR "three dimensional printing":ti,ab,kw OR "additive manufacturing":ti,ab,kw OR "patient-specific":ti,ab,kw OR "custom-made":ti,ab,kw OR customized:ti,ab,kw) AND ("vertebral body replacement":ti,ab,kw OR "vertebral replacement":ti,ab,kw OR VBR:ti,ab,kw OR "artificial vertebral body":ti,ab,kw OR AVB:ti,ab,kw OR prosthes*:ti,ab,kw OR implant*:ti,ab,kw OR cage*:ti,ab,kw) AND ("total en bloc spondylectomy":ti,ab,kw OR TES:ti,ab,kw OR spondylectom*:ti,ab,kw OR "en bloc":ti,ab,kw OR vertebrectom*:ti,ab,kw OR corpectom*:ti,ab,kw OR resect*:ti,ab,kw OR reconstruct*:ti,ab,kw) AND [1980-2026]
