# Supplementary figures and images for: Complications and Revision Patterns After 3D-Printed Vertebral Body Replacement for Spinal Tumors: A Systematic Review and Critical Appraisal
Source: J Clin Med. 2026 Apr 30;15(9):3447. doi: 10.3390/jcm15093447 (PMC13164235; doi:10.3390/jcm15093447)

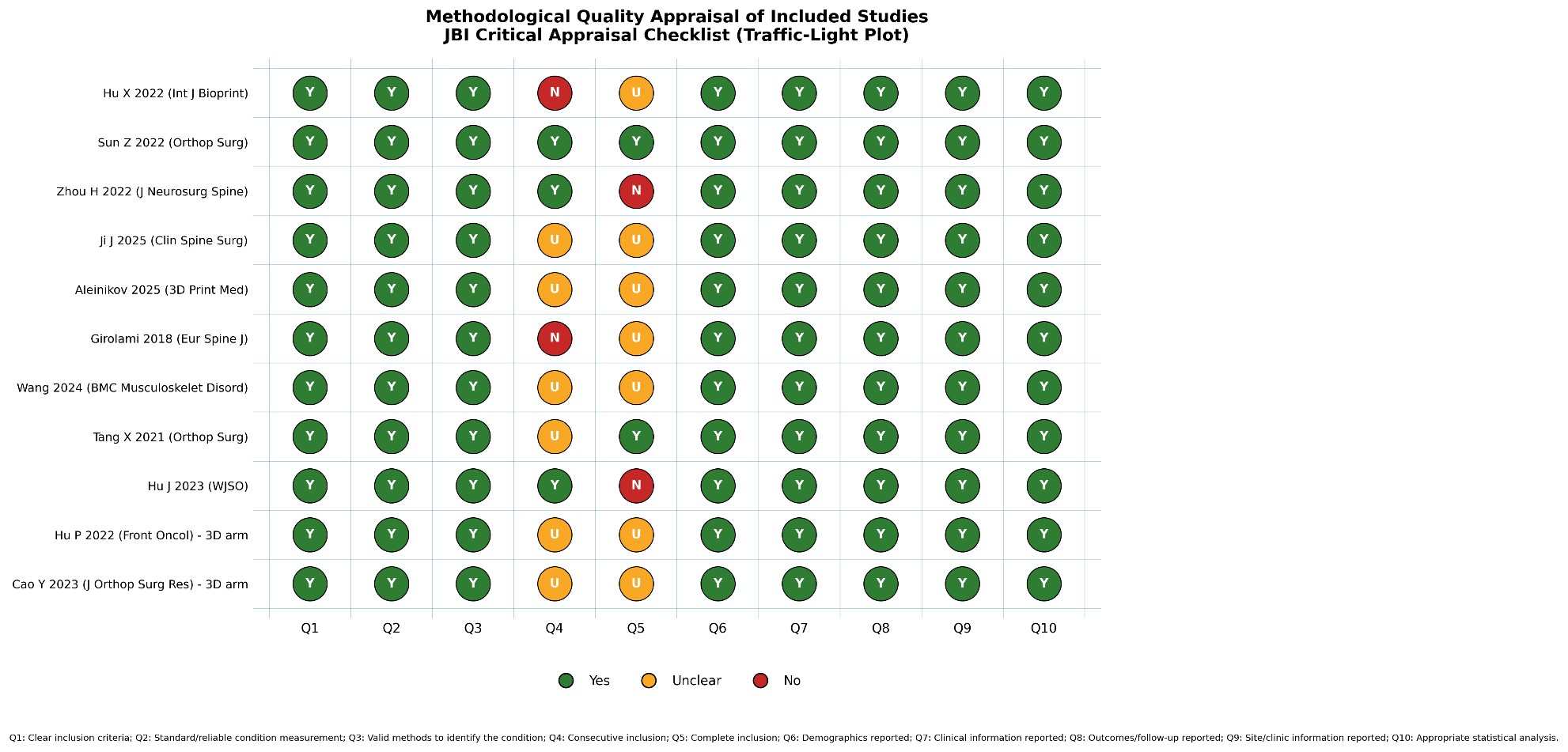

Supplement: Supplementary file 1 [file jcm-15-03447-s001.zip › figure s1.jpg]
